# New Aerodynamic Studies of an Adaptive Winglet Application on the Regional Jet CRJ700

**DOI:** 10.3390/biomimetics6040054

**Published:** 2021-09-24

**Authors:** Marine Segui, Federico R. Abel, Ruxandra M. Botez, Alessandro Ceruti

**Affiliations:** 1 Laboratory of Applied Research in Active Controls, Avionics and AeroServoElasticity (LARCASE), École de Technologie Supérieure, Montréal, QC H3C1K3, Canada; marine.segui.1@ens.etsmtl.ca; 2 Department of Industrial Engineering, University of Bologna, 40136 Bologna, Italy; federico.abel@studio.unibo.it (F.R.A.); alessandro.ceruti@unibo.it (A.C.)

**Keywords:** morphing, adaptive, winglet, aerodynamic, optimization, longitudinal, subsonic, transonic, OpenFoam

## Abstract

This study aims to evaluates how an adaptive winglet during flight can improve aircraft aerodynamic characteristics of the CRJ700. The aircraft geometry was slightly modified to integrate a one-rotation axis adaptive winglet. Aerodynamic characteristics of the new adaptive design were computed using a validated high-fidelity aerodynamic model developed with the open-source code OpenFoam. The aerodynamic model successively uses the two solvers *simpleFoam* and *rhoSimpleFoam* based on Reynold Averaged Navier Stokes equations. Characteristics of the adaptive winglet design were studied for 16 flight conditions, representative of climb and cruise usually considered by the CRJ700. The adaptive winglet can increase the lift-to-drag ratio by up to 6.10% and reduce the drag coefficient by up to 2.65%. This study also compared the aerodynamic polar and pitching moment coefficients variations of the Bombardier CRJ700 equipped with an adaptive versus a fixed winglet.

## 1. Introduction

With the substantial increase in carbon dioxide (CO_2_) emissions into the atmosphere over the past several years [1,2], the aerospace sector has to limit its figures in the near future [3,4]. For that, some solutions are available, such as working on trajectories optimization [5,6,7,8,9,10] or improving aircraft models to design reliable flight simulators [11,12,13,14]. It has been shown that trajectory optimization can save up to 2% of the cost of flights. Moreover, the use of reliable flight simulators could replace real flights related to pilot training or aircraft launch (certification, optimizations, etc.) and, consequently, could also help to reduce CO_2_ emissions. To complement these efforts, solutions involving the optimization of aircraft geometry could also be interesting.

### 1.1. Literature Review: Aircraft Geometry Improvement

Other solutions aim to improve the aircraft geometry and reduce the drag as a means to reduce fuel consumption [15,16,17,18]. Among the notable advances, the arrangement of a winglet at the wingtip is considered as one of the most important. When an aircraft is flying, the high-pressure flow (below the wing) joins the low-pressure flow (over the wing) by generating a vortex and therefore creating undesired drag. By introducing a winglet device at the wingtip, Whitcomb improved the cruise efficiency from 6% to 9% and increased the mileage by 6.5% for a National Aeronautics and Space Administration (NASA) test aircraft [19]. Various winglet shapes have been developed, such as the blended winglet [20], wingtip fences [21], split-scimitar winglets [22], and raked wingtips [23]. Each shape has its own advantage, as, for example, a blended winglet offers a reduction of the fuel consumption from 4 to 5% depending on the aircraft type [20].

The latest winglet (or wingtip) configuration is the one that equips the Boeing 777X aircraft [24]. Developed with an unconventional wingspan that optimizes its performance, the Boeing 777X is code F airport compatible (its wingspan is from 65 m to 80 m); therefore, it can only access F-equipped airports (limited to roughly 400 airports worldwide). To expand its scope, Boeing engineers have developed a folding wingtip for use in ground phases. Indeed, by folding 3.5 m of the wing on each side, the Boeing 777X can operate on the ground as most conventional long-range aircrafts (code E airport compatibility) [25]. According to the Federal Aviation Administration (FAA), to be certified, this device must be completely folded for taxi phases and unfolded for flight phases [26]. However, it would be interesting to measure how the Boeing 777X performances can be influenced by the fact the wingtip will also move (under control) during flight. Furthermore, the winglet mechanism is already installed; making it active during the flight will not add any significant weight at the wing tip.

Some studies have been done already under the name of “adaptive winglet”, “morphing winglet”, or “active winglet”, and these studies have indicated promising ideas. Among these studies, the project CLAReT (Control and Alleviation of Loads in Advanced Regional Turbo-Fan Configurations) was conducted in 2013 by the Clean Sky team and Bristol university [27]. CLAReT researchers developed a “morphing winglet concept” that aims to adapt the winglet cant (i.e., dihedral) and twist angles throughout the flight envelope of a regional jet. For the three speed conditions studied, Mach numbers 0.48, 0.60, and 0.74, the aircraft operational range was increased by up to 5%. To achieve these results, the CLAReT team conducted a structural analysis and aerodynamic studies using Computational Fluid Dynamics (CFD) methods [28,29]. Bristol university and the aircraft manufacturer Airbus have also conducted a “free winglet” project called “AlbatrossOne”, able to reduce bending moments absorbed by the wing during the flight, and therefore, it could lead to a weight reduction [30,31,32].

Teams from Airbus [33] and the French aerospace laboratory (ONERA) [34,35] are also working on an adaptive winglet applied to a regional aircraft. The device imagined is in fact a new control surface integrated into the trailing edge of a conventional winglet. In other words, the winglet is morphed according to its camber during the flight. Researchers have presented an important work regarding the mechanism and structural aspects, but to date, no significant aerodynamic results were obtained [36].

The state-of-the-art also offers a “telescopic” winglet that consists of an enlargement or a shrinking of the winglet span. With this device, researchers have successfully reduced the fuel consumption of a transport jet aircraft similar to the Airbus A380 by up to 2% based on the Breguet formula and CFD aerodynamic computations [37].

Contributing to the work on global warming, recent studies performed by the Laboratory of Applied Research in Active Controls, Avionics, and AeroServoElasticity (LARCASE) were targeted to improve aircraft aerodynamics characteristics using adaptive structures (i.e., “morphing wings” or “adaptive wings”) [38]. The knowledge and skills of LARCASE in this discipline were notably highlighted in 2014 by winning the Consortium for Research and Innovation in Aerospace in Québec (CRIAQ) runner-up award for the CRIAQ MDO505 project, entitled “Morphing Architectures and related Technologies to improve the Wings Efficiency” [39,40,41,42,43,44,45,46,47].

In 2018, the LARCASE team conducted a preliminary study to measure the impact of an adaptive winglet for a Cessna Citation X [48,49]. This study used Vortex-Lattice Method (VLM) aerodynamic computations to measure the aerodynamic characteristics of the aircraft in its flight envelope [48,49]. In addition, an in-house performance model of the aircraft has shown that rate of climb could be increased by up to 26 feet/min during climb segments and fuel flow reduced by up to 20 pounds (9.1 kg) per hour for cruise segments, using an adaptive winglet. With such promising results, the authors recommended to pursue this study and use Computational Fluid Dynamics (CFD) based on Navier–Stokes equations in order to confirm the results obtained using VLM [48,49]. Indeed, VLM can predict induced drag (without compressibility effects) but not skin friction and shape (called also “pressure” or “form”) drag. Turbulence effects are also neglected with a VLM method.

### 1.2. Paper Objectives

The literature shows that numerous research has been interested in adaptive winglet structural studies, but only a few studies have measured aerodynamic characteristics. Similarly, the literature lacks studies which would lead to predict the performance inflight of an aircraft equipped with an adaptive system.

The goal of this research is to continue the work realized using the VLM methods by the LARCASE team [48,49]. The adaptive winglet design has been investigated in terms of a complete aerodynamic study over the whole flight envelope of a regional jet. A further study will then evaluate the benefits of an adaptive winglet in terms of “flight performance”; for this reason, only longitudinal aerodynamic characteristics were studied in this paper. As structural data of the aircraft is not available, mechanical and aero elastic aspects were not studied in this paper. However, this study should highlight all the information to further design the winglet motion mechanism.

This research will evaluate the aerodynamic efficiency of an adaptive winglet in flight with respect to a fixed (i.e., conventional) winglet. Among the benefits considered, a winglet is one of the last parts assembled onto an aircraft wing, and thus, it can be quite easily replaced on a fleet. Given that the surface of the winglet would need to be controllable, certification phases would be necessary, but these phases would be shorter and less expensive than for a new aircraft certification. 

Adaptive winglets could therefore present a significant ecological aspect, as they could allow the improvement of aircraft performance already on the market, without the need for replacing them. Moreover, by applying this winglet change to a whole fleet, the International Civil Aviation Organization (ICAO) objectives could be realized in a relatively short time, while limiting the related expenses.

To perform this present study, the Bombardier CRJ700 [50] was chosen because the LARCASE has a high-quality flight simulator for this aircraft. The Bombardier CRJ700 flight simulator located at LARCASE, called the Virtual Research Simulator (VRESIM), is a simulator designed and assembled by CAE Inc. for the research needs of LARCASE.

The VRESIM is a level D flight simulator that is able to simulate flight test data with less than 5% of error with respect to those of the real aircraft [51]. Indeed, the level D is the highest degree delivered by the Federal Aviation Administration (FAA) to qualify the flight dynamics of a simulator. In addition, aerodynamic data provided by the VRESIM was simulated based on original aerodynamic data tables delivered by the aircraft designer Bombardier for the CRJ700. In consequence, the VRESIM is a very reliable source for validation.

## 2. Methodology: Adaptive Winglet Analysis

The methodology aims to quantify the advantages and disadvantages of equipping an aircraft with an adaptive winglet from an aerodynamic point of view. For this purpose, an adaptive winglet was designed for the CRJ700 aircraft. Then, a high-fidelity aerodynamic model of the aircraft was used to compute lift, drag, and pitching moment forces for several flight conditions and several winglet deflection angles.

### 2.1. Adaptive Winglet Design

The adaptive winglet considered in this research was designed based on the original winglet of the CRJ700. Winglets that equipped the original Bombardier CRJ700 are commonly called “canted winglets”. As a signature of Bombardier aircraft, “canted winglets” have a curved part at the trailing edge. 

Due to the mechanical difficulties involved in moving the original winglet showing a thin thickness (0.15 m at the root), we decided to slightly modify the original shape of the winglet by incorporating a “pod” at its root. The use of this component shape was inspired by the Airbus Blade project [52] and the Boeing *777X* winglet motion [24].

The pod’s exposed surface is 0.654 m^2^, its length is 1.372 m, and its maximum diameter is 0.173 m. It was designed to allow enough space inside of the pod to install a gear mechanism or a similar actuation device, as in the case of the Boeing *777X* winglet motion. Given that this study is targeted at aerodynamic analysis and, further, performance benefits, the hypothesis that this pod could accommodate an adequate mechanism using current technology was made. 

**Figure 1:**
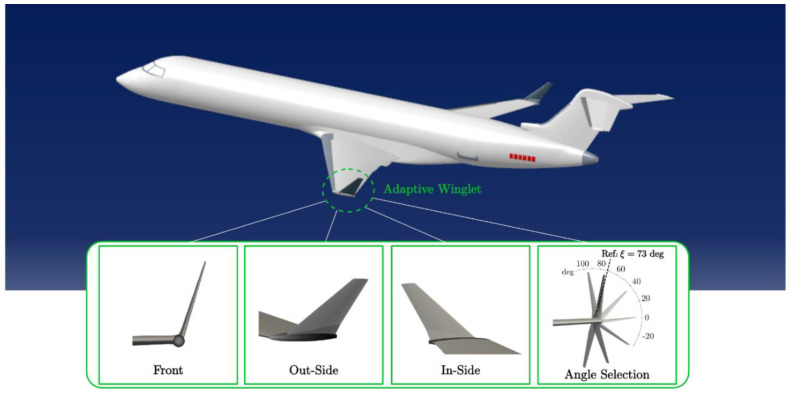
Bombardier CRJ700 adaptive winglet.

Figure 1 shows the Bombardier CRJ700 equipped with the adaptive winglet. It can be observed that the winglet of this proposed aircraft configuration has the capability of moving following one rotation axis. When the adaptive winglet makes an angle ξ of 73 deg, it can be superimposed onto the original Bombardier winglet. The winglet angle ξ is measured between the winglet axis and the ground.

### 2.2. Presentation of the Aerodynamic Model

In order to analyze the new aerodynamic characteristics brought by the adaptive aspect of the winglet, a high-fidelity aerodynamic model was used as recommended by previous work [48,49]. The method here consisted in using the aerodynamic model designed and validated for the original Bombardier CRJ700 to perform simulations of the aircraft equipped with the adaptive winglet. As this model was developed in a previous study, all details and design justifications, such as mesh analysis or turbulence model choice, were not covered in this paper. For more details, please refer to the work that contributed in developing the original aerodynamic model of the CRJ700 [53].

The aerodynamic model of the Bombardier CRJ700 used in this study was implemented using the open-source CFD code OpenFoam (i.e., mesh and simulations utilities) in such a way as to limit computation expenses. Indeed, computers that have limited computation capacity were used to design the mesh and perform computations (computers equipped with eight processors and 32 GB of RAM memory). Meshes were designed as coarse as possible while providing acceptable qualities. In addition, a new simulation process consisting of using two successive solvers was developed in order to optimize solution convergence and stability (i.e., especially required for coarse/medium mesh).

#### 2.2.1. Mesh Design

**Figure 2:**
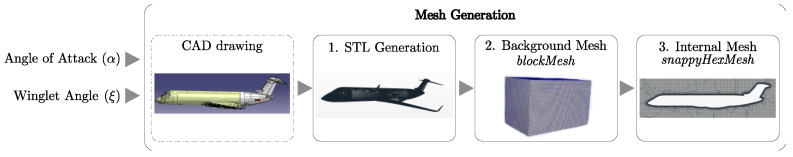
Mesh design outlines.

The mesh of the aerodynamic model was designed using *blockMesh* and *snappyHexMesh* OpenFoam utilities (Figure 2). For simulations using an angle of attack different from zero, it was preferred to rotate the aircraft instead of the flow. In consequence, one mesh was developed for each combination of the angle of attack (from −2 degrees to 4 degrees) and the winglet deflection angle (for the adaptive winglet version of the aircraft).

The aircraft shape (i.e., including angle of attack and winglet deflection angle) was designated using a triangulated file (STL) from the CAD model (Figure 1). The *blockMesh* utility was used to design the “background mesh”, which is a domain of 44 m × 44 m × 70 m composed of hexahedral cells of 1 m^3^ (1 × 1 × 1 m). Then, the *snappyHexMesh* (*SHM*) tool was used to produce the aircraft integration (i.e., the aircraft shape defined using the STL file) in the “background mesh”. In order to perfectly smooth the wall of the plane, *SHM* use an algorithm that made refinement, smoothing, and re-alignment treatments using several techniques, such as chimera technics and curvilinear mesh [53,54]. The *SHM* algorithm performs mesh re-arrangement until the mesh quality criteria have been reached. In this study, the maximum non-orthogonality was set to 65, and the maximum skewness was set to 5. The refinement near wall was set using three layers measuring a maximum of 8.5 mm in total. The first layer, the closest to the wall, did not exceed 1.55 mm. 

Meshes obtained for the original CRJ700 design were composed on average by 11.14 × 10^6^ cells, and they showed a y+ value between 100 and 200, which has been considered as a “medium” mesh.

#### 2.2.2. Simulation Settings

The CRJ700 flies up to Mach number 0.85; in consequence, a compressible solver is required in order to consider the compressibility effect [55]. Moreover, the model was designed to study the CRJ700 in steady-state situations, and therefore, it exploited solvers using time-averaged Navier–Stokes equations (RANS) [54].

**Figure 3:**
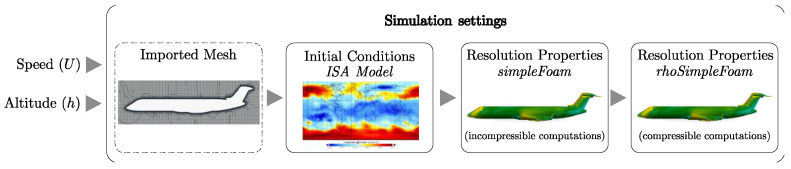
Simulation process summary.

As meshes were designed as coarse as possible, while keeping an acceptable quality level due to computer resources limitations, it was necessary to take these aspects into account for the simulations steps. Indeed, with the “medium” mesh designed, it was difficult to reach a good rate of convergence using a compressible solver (such as *rhoSimpleFoam*, for instance). In order to avoid this problem, a special process was developed for the CRJ700 aerodynamic model. It consists of using two successive OpenFoam solvers, as illustrated in Figure 3. We observed that instability in solutions has been avoided during design and validation studies of the aerodynamic model [53]. 

A first estimation of the flow properties was computed using the incompressible solver *simpleFoam*, initialized using atmospheric data issued from an International Standard Atmosphere (ISA) model. ISA equations give the theoretical properties of an atmospheric flow in terms of its pressure, air density, dynamic viscosity, temperature, etc., according to the altitude (without any temperature deviations). 

The converged solution obtained with *simpleFoam* (the first simulation) was then used to initialize the flow for the compressible solver *rhoSimpleFoam* (density-based) [53]. This second simulation is “stable”, especially due to the fact that it was initialized using a converged solution of pressure and speed flow. New parameters that were necessary to be estimated, such as turbulent parameters and the temperature, were then predicted in a more stable way than without a “pre-simulation” (i.e., *simpleFoam*).

Concerning the choice of turbulence model, it was found that coupling the solver *simpleFoam* with the Spalart–Allmaras (S-A) turbulence model was the most stable option, especially due to its one-equation representation. For the second simulation, where accuracy was more important than computation stability (because stability was maintained due to flow initialization), the turbulence model k−ω Shear Stress Transport (SST) was selected [53].

#### 2.2.3. Validation of the CRJ700 Aerodynamic Model

**Figure 4:**
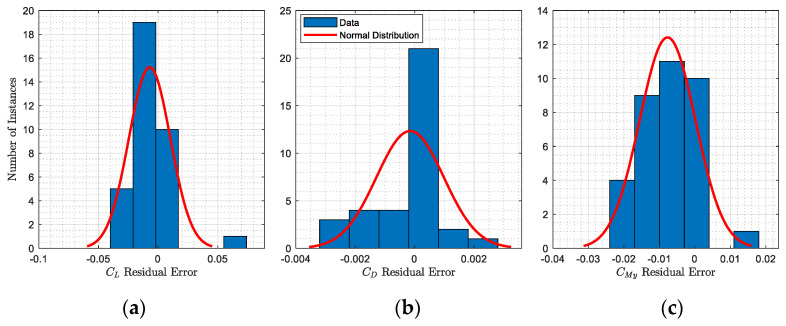
Statistical analysis of errors obtained between the reference (VRESIM) and the validated aerodynamic model of the CRJ700 using OpenFoam. Residual errors are presented in terms of lift (**a**), drag (**b**), and pitching moment (**c**) coefficients.

Finally, the aerodynamic model was tested for 35 flight conditions frequently flown by the Bombardier CRJ700, for which the altitude ranges from 5000 ft to 30,000 ft, the Mach number from M0.31 to M0.79 (normal cruises are operated between M0.74 and M0.82), and the angle of attack from −2 degrees to 4 degrees. Figure 4 indicates the statistical study of the errors obtained between aerodynamic coefficients computed by the OpenFoam model versus the reference (i.e., VRESIM). Figure 4a–c show the results in terms of lift, drag, and pitching moment coefficients absolute errors, respectively.

Lift coefficients of the CRJ00 were predicted by the aerodynamic model design within an error margin of −0.007 ± 0.045 in 95% of cases. Similarly, the drag and the pitching moment coefficients were estimated within error margins of −0.00015 ± 0.00114 and −0.0077 ± 0.0079 in 95% of cases, respectively. Based on these low errors (Figure 4), it was assumed that the aerodynamic model of the Bombardier CRJ700 designed using OpenFoam was highly accurate, and it had therefore been validated to perform the adaptive winglet study [53].

By considering the validated aerodynamic model as accurate, all its properties, hypotheses, and settings were re-used to compute the aerodynamic characteristics of the adaptive configuration of the airplane (i.e., equipped with the adaptive winglet) by changing the input geometry. 

### 2.3. Comparison of the Original and the Adaptive Winglet Designs of the CRJ700

This section aims to compare the original and the adaptive winglet design of the CRJ700. Indeed, to design the adaptive winglet version of the CRJ700, some modifications were made at the winglet level. To use the CRJ700 validated aerodynamic model to study the characteristics of the aircraft equipped with the adaptive winglet, the context, such as mesh qualities, must remain similar. 

Indeed, similar mesh qualities allow to use the same solver settings as those used for the validation of the aerodynamic model. In this context, the same level of confidence (of the validated model) can be expected in the results of the simulations. 

#### 2.3.1. Geometric Comparison

**Figure 5:**
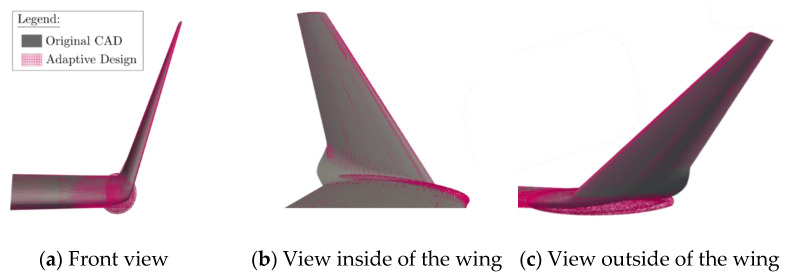
Superposition of the Computer-Aided-Design (CAD) of the original model and the new adaptive model of the CRJ700 aircraft.

Firstly, a geometric comparison was made in Figure 5. The original winglet shape that equipped the Bombardier CRJ700 is represented in a “dark grey” color, and the adaptive winglet design is shown in a “pink” color. 

From Figure 5, it can be observed that the original and the new adaptive designs are very similar. The main difference is located around the winglet root with the addition of the pod. Indeed, the pod is slightly bigger than the original winglet at the junction between the wing and the winglet. 

As the adaptive winglet geometry is larger than the original winglet geometry, it was expected that this new design, including the pod, would slightly increase the drag. 

#### 2.3.2. Aerodynamic Comparison

Firstly, the mesh qualities obtained for both aerodynamic models (for the validated and for the new adaptive model of the CRJ700 aircraft) were compared and described in Table 1 in terms of cell’s “maximum non-orthogonality”, “maximum skewness”, and “number of cells”. 

As a reminder, one mesh was generated per angle of attack studied using the validated model, which represents seven meshes. The average qualities of these seven meshes were added on the second column of Table 1. Concerning the new aerodynamic model (to study the adaptive winglet), as the winglet deflection angle would change, a new mesh is also needed for each winglet deflection angle. In order to check the validity of meshes generated for the new adaptive design, it was decided to analyze meshes generated for the combination of the seven angles of attack and the winglet deflection angles ξ={−93,−73,−35, 0, 35, 73 and 93} deg. Averaged meshes qualities obtained for these 49 meshes (for the new adaptive design model) are displayed in the third column of Table 1.

Globally, the figures displayed in Table 1 reveal that all the meshes have qualities in the same order of magnitude. For the “maximum non-orthogonality” criteria, it was observed that 65.06 deg (for the validated model) and 65.02 deg (for the new model) are extremely close. Similarly, cells’ maximum skewness values were also very close, such as 5.00 for the validated model and 4.99 for the new model that includes pod design. In addition, meshes were designed using a similar number of cells: close to 11.14 × 10^6^ cells for validations cases and using approximatively 11.12 × 10^6^ cells for adaptive cases (equipped with the pod). Therefore, it seems that the “pod” designed in order to integrate the adaptive winglet was not linked to a degradation of the mesh qualities, which is very favorable to pursue the study. 

As the meshes have shown very similar qualities to those of the original model, the same solver (*simpleFoam* and *rhoSimpleFoam*) settings as those used for the validated aerodynamic model were kept.

As a second verification study, the aerodynamic characteristics of the original (without a pod) and the adaptive (equipped with a pod and a winglet fixed and set at angle ξ= 73 deg) aircraft configuration of the CRJ700 were compared in order to assess how the pod affects the aerodynamic coefficients values. 

**Figure 6:**
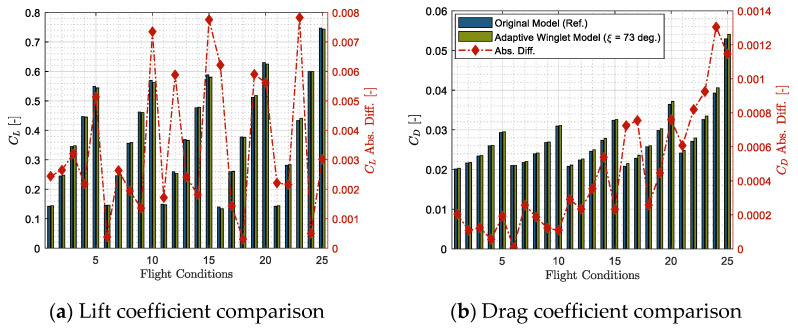
Comparison of aerodynamic coefficients obtained using the aerodynamic model for the original and the adaptive winglet design of the CRJ700 Aircraft.

Figure 6 shows the lift and drag coefficients delivered from the aerodynamic model for the original CRJ700 design (data validated with the VRESIM) and for the adaptive winglet configuration of the aircraft. These results are given for 25 different flight conditions for which the aerodynamic model has been validated. 

As the differences in terms of pitching moments are very small (0.0031 on average), it was considered that the pod does not have a high influence on this aerodynamic characteristic. Generally, the results of both models are “close enough” (Figure 6a,b), which indicates that the aerodynamic model used for both models correctly computes the aerodynamic performance of both aircraft designs. 

It is important to clarify that it is normal that the two models do not overlap, as it is not exactly the same aircraft being considered (one has the original Bombardier CRJ700 winglet, in “blue” color, and the other has an adaptive winglet with a pod, arranged at 73 deg, in “green” color). It can be observed in Figure 6a,b that there is a “gap” of error between the aerodynamic characteristics of the two models which reflects the impact of the pod integration.

Depending on the flight condition, the pod seems to have a different impact on the lift coefficient (Figure 6a). Indeed, sometimes, the pod induced an increase on the lift coefficient, and sometimes a decrease, but the difference was always less than 0.008, which is very small. This non-constant trend seems to be determined by the turbulence computation, as its variation is very complex according to flight conditions.

The drag coefficient (Figure 6b) for the adaptive winglet configuration (in “green” color) is higher than the drag coefficient obtained for the original design (in “blue” color) by 0.0005 on average. It was expected that the drag would be larger for the adaptive winglet configuration, because the pod is larger than the original winglet at the root level and, thus, it induces higher drag. Moreover, we noticed that the drag coefficient error increases as the Mach number increases.

This section has highlighted that the adaptive winglet design does not have a strong impact on the mesh qualities nor on the aerodynamic characteristics. Therefore, the same solution settings of the validated aerodynamic model can be kept, and the same level of accuracy can be expected to compute characteristics of the new adaptive design. 

However, small differences were observed from the aerodynamic point of view, especially due to the “pod” integration in the design. In order to carefully analyze the benefit of the adaptive winglet compared to a fixed one, it was decided to define that the reference of the study should be the adaptive winglet design set with a deflection angle of 73 deg. This choice allows to consider the pod disadvantages of the adaptive winglet design while highlighted the adaptive aspects of the winglet. 

It is important to emphasize that the new junction (i.e., the “pod”) was not geometrically optimized in this first study. Indeed, as this research aims to measure the advantages of an adaptive winglet versus those of a fixed winglet, it was preferred to compare the aerodynamic characteristics of two versions of the “same” winglet (one version fixed at 73 deg and one version able to move). Therefore, if an improvement was observed using this adaptive winglet, an even greater improvement was expected in the case when an optimized junction was implemented.

### 2.4. Aerodynamic Simulations

Aerodynamic characteristics of the new adaptive design of the CRJ700 were computed using the validated aerodynamic model presented in Section 2.2.

To study the widest range of the winglet deflection angle, it was assumed that the adaptive winglet limits its angle ξ variation between −93 deg and 93 deg. Since conducting CFD analysis in such a wide range can be time consuming, the strategy consists in studying the aerodynamic characteristics for specific winglet positions and then performing a continuity analysis to predict aerodynamic characteristics between these positions.

#### 2.4.1. Aerodynamic Simulations for Specific Winglet Deflection Angles

The aerodynamic characteristics of the Bombardier CRJ700 equipped with the different winglet deflection angles ξ={−93,−73,−35, 0, 35, 73 and 93} deg were evaluated for various flight conditions, the most representative of the whole flight envelope of the aircraft. This range of winglet deflection angle was chosen arbitrarily, except for ξ= 73 deg, which was considered as the reference angle.

Flight conditions choices were achieved by performing a wide range of flight tests (climb, cruise, and descent segments) on the whole flight envelope using the VRESIM flight simulator. Moreover, only “clean” configurations of the aircraft (i.e., no slats, no flaps, and no gears) were considered.

The flight conditions selection was made from conditions often used during flight tests, as well as according to flight conditions that were validated using the original aerodynamic model of the CRJ700. A recap of the tested flight conditions is presented in Table 2.

For each set of flight conditions (combinations of an altitude, Mach number, and angle of attack) described in Table 2, winglet positions ξ={−93,−73,−35, 0, 35, 73 and 93} were studied. It is important to add that for all flight conditions simulated, the horizontal tail of the CRJ700 aircraft was set at its neutral position (0 deg).

#### 2.4.2. Continuity Study

As a second step, a continuity study was required to predict values of the lift, drag and pitching moment coefficients between the selected flight conditions (Table 2) and between two positions of winglet deflection angles ξ studied (i.e., the range: {−93,−73,−35, 0, 35, 73 and 93}). 

**Figure 7:**
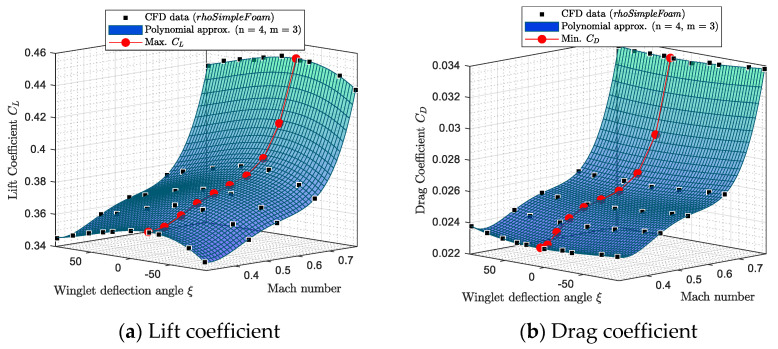
Lift and drag coefficients variations with the winglet deflection angle and Mach number for an angle of attack of 0 deg.

**Figure 8:**
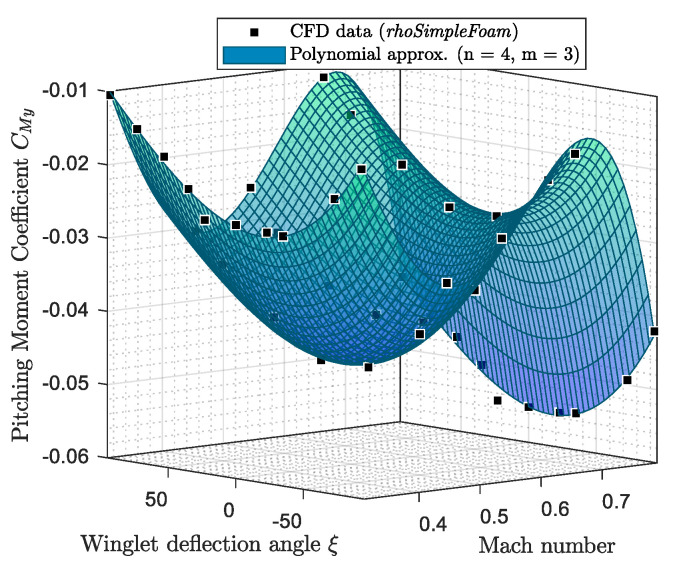
Pitching moment coefficient variation versus the winglet deflection angle and Mach number for an angle of attack of 0 deg.

The variation of the aerodynamic coefficients ($C_{L},\;C_{D},\;\mathrm{and}\;C_{My}$) with the Mach number and winglet deflection angle ξ, for a fixed angle of attack, was analyzed. Figure 7 and Figure 8 show the variation of the aerodynamic coefficients ($C_{L},\;C_{D},\;\mathrm{and}\;C_{My}$) when α = 0 deg; the winglet deflection angle varies from −93 deg to 93 deg, and the Mach number changes from 0.31 to 0.79. 

Aerodynamic coefficients obtained by *rhoSimpleFoam* CFD simulations are represented using black squares on Figure 7 and Figure 8. Then, these graphs show a fitting polynomial surface that was investigated in order to determine the variation of aerodynamic coefficients between CFD measures. 

For the three longitudinal aerodynamic coefficients, the fitting surfaces with the closest R^2^ to 1 (R^2^ > 0.96) were polynomial surfaces corresponding to a fourth order for the Mach number inputs and to a third order for the winglet deflection angle inputs. As a reminder, R is the correlation factor usually used in identification methods, such as linear regression [56].

The polynomial fitting surface equations for $C_{L},\;C_{D},\;\mathrm{and}\;C_{My}$ are presented in Equation (1), where $a_{i,j}$ are polynomial coefficients, and *M* and ξ designate the Mach number and the winglet deflection angle, respectively.

(1) $$C_{L,\;D,\;M_{y}}\left(M,\;\xi \right)=\overset{n=4}{\underset{i=0}{\sum }}\overset{m=3}{\underset{j=0}{\sum }}a_{ij}\cdot \;M^{i}\cdot \;\xi^{j}$$

It is important to add that due to the fact that an order of three was necessary to model aerodynamic coefficient surfaces versus the winglet deflection angle, it means that the variation of aerodynamic coefficients ($C_{L},\;C_{D},\;\mathrm{and}\;C_{My}$) is non-symmetrical on both sides of the 0 deg winglet position. This non-symmetrical behavior is attributed to the winglet planform, which originally had a very small camber in the adaptive winglet design. It could also be attributed to the loading distributions changes, particularly as the effective span changes (i.e., the aspect ratio).

Using fitting surfaces, the winglet deflection angles corresponding to the maximum value of $C_{L}$ and minimum values of $C_{D}$ for each Mach number were analyzed. These results are displayed in Figure 7a,b, using “red” dots. Figure 7a shows that the winglet deflection angle that corresponds to the maximum of $C_{L}$ depends on the Mach number. In other words, the winglet deflection angle that gives the maximum of $C_{L}$ is a function of the Mach number (for a fixed angle of attack). This observation can also be done for the minimum value of $C_{D}$ in Figure 7b. However, the winglet deflection angle that corresponds to the maximum value of $C_{L}$ is not necessarily the same that offers the minimum value of $C_{D}$ (for a given Mach number and angle of attack). 

## 3. Results

This third section presents the results obtained for the aerodynamic study of an adaptive winglet application for the Bombardier Regional Jet CRJ 700. 

### 3.1. Aerodynamic Benefits of an Adaptive Winglet

In order to highlight the benefits of an adaptive winglet, it is important to select an optimization criterion. Usually, the optimization criteria concern inflight parameters, such as a flight time or the fuel consumption. Since this study deals only with the aerodynamic aspects, improvement performed using an adaptive winglet do not allow one to conclude on the final performances that could be offered during flight.

**Figure 9:**
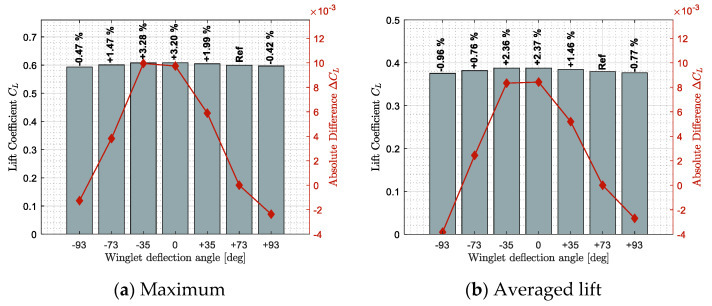
Maximum and averaged lift benefits observed for different winglet deflection angles and different flight conditions.

**Figure 10:**
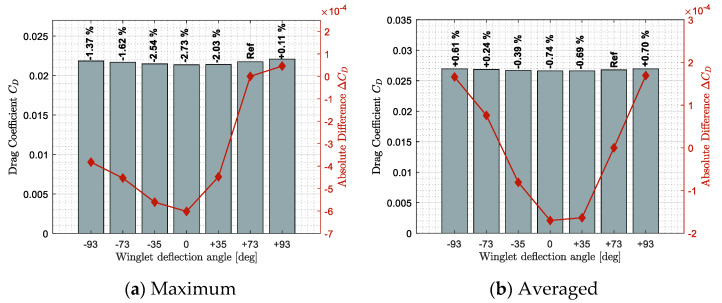
Maximum and averaged drag benefits (reduction) observed for different winglet deflection angles and different flight conditions.

**Figure 11:**
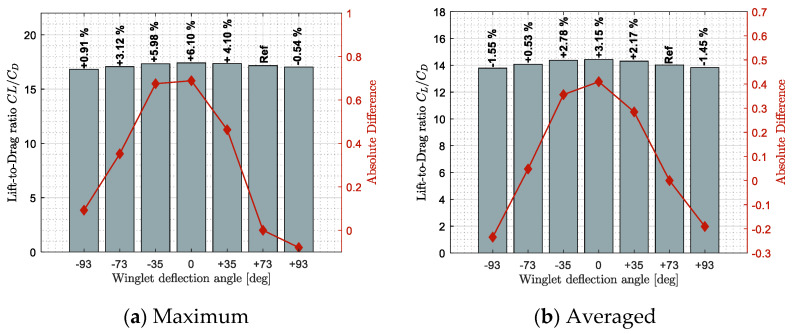
Maximum and averaged benefits observed on the lift-to-drag ratio for different winglet deflection angles and different flight conditions.

Figure 9, Figure 10 and Figure 11 show benefits observed in terms of lift, drag, and lift-to-drag ratio, respectively. These results highlight how a specific winglet deflection position ξ should improve the fixed winglet configuration (set at ξ = 73 deg). Benefits were computed using two statistical tools, the maximum benefit value on sub-figures (a) and the averaged benefit value on sub-figures (b), obtained among all the flight conditions tested.

Figure 9 shows the lift (left axis) and the lift difference (right axis) obtained for each winglet deflection angle from ξ = −93 to 93 deg. As additional information, the relative difference obtained between a specific winglet deflection and the reference position was displayed above the corresponding bars. 

In Figure 9, the negative difference obtained for winglet deflection angles ξ = −93 deg and ξ = +93 deg can be observed. This demonstrates that ξ = ± 93 deg degrades (i.e., reduce) the lift coefficient with respect to the reference position (ξ = 73 deg). On the other hand, other winglet deflection angles (ξ = −73 deg to ξ = +35 deg) have shown a lift benefit (i.e., increase) with respect to the reference position. Indeed, a maximum lift increase of 3.28% have been obtained for the winglet position ξ = −35 deg (Figure 9a). Similarly, a winglet position ξ = 0 deg (close to horizontal) has shown an average lift improvement of 2.37% (Figure 9b).

Concerning the drag, it was noticed that the different winglet deflection angles could reduce the drag (i.e., benefit) differently. For specific flight conditions that were conducted towards the maximum drag improvement (minimization) (Figure 10a), it was shown that all winglet deflection except the position ξ = +93 deg showed a drag reduction from 1.37% to 2.73%. However, it was observed that only winglet deflection angles ξ = −35 deg to +35 deg could reduce the drag coefficient of the aircraft on average for all the flight conditions tested (Figure 10b). This observation allows us to highlight the advantage of an adaptive winglet. Indeed, for specific flight conditions (such as the ones that offered the maximum drag difference in Figure 10a), certain winglet positions can offer a really advantageous aerodynamic performance, such as a drag reduction of up to 2.73%.

The last aerodynamic criterion is the lift-to-drag ratio. As some advantages of an adaptive winglet in terms of lift and drag coefficients were observed, good improvements were also expected in terms of the lift-to-drag ratio in Figure 11. Since the improvement of using an adaptive winglet in terms of lift coefficient was higher than the improvement in terms of drag, the lift-to-drag results follow a similar trend as the lift results (Figure 9).

By this way, on average, winglet deflection angles from ξ = −73 deg to +35 deg showed a lift-to-drag increase (i.e., benefit) of up to 3.15% (Figure 11b). For specific flight conditions, it was observed that the winglet position ξ = −93 deg could increase the lift-to-drag ratio by up to 0.91% (Figure 11a). Similarly, for specific flight conditions, other winglet deflection positions could increase the lift-to-drag value by up to 6.10%.

Results shown on Figure 9, Figure 10 and Figure 11 have shown the advantage of using an adaptive winglet, as different winglet deflection positions could offer lift, drag, or lift-to-drag ratio improvement with respect to the reference configuration of the aircraft. Generally, winglet positions ξ = ± 93 deg offered the “worst improvement”, and in reverse, winglet positions ξ = −35 deg to ξ = +35 deg showed the best aerodynamic characteristics. 

Most of the best improvements were corresponding to the winglet deflection position of ξ = 0 deg, which may affect the choice of using an “adaptive” winglet instead of a fixed one set at ξ = 0 deg. This result was expected, as the position ξ = 0 deg corresponds to the highest aspect ratio of the wing, and consequently, it is the “ideal” position to increase the lift and reduce the induced drag. As a reminder, usually, the pilot needs to fly at different optimum criterion, such as the maximum speed, the minimum flight time, etc., which are not necessarily corresponding to an optimal aerodynamic criterion, such as lift, drag, or lift-to-drag ratio (all along the flight). 

In this study, using aerodynamic criteria, we have shown that the adaptive winglet allows for the improvement of aerodynamic characteristics for several flight conditions thanks to its adaptive aspect. Consequently, it could be expected that during the flight, the “optimal” winglet deflection position should be different from the *ξ* = 0 deg position, as the optimal flight criteria should be based on aircraft performances instead of aerodynamic characteristics.

### 3.2. Comparison of the Characteristics of the CRJ700 Equipped with Fixed versus Adaptive Winglets in Terms of Aerodynamic Polar and Pitching Moment 

Based on the optimization criteria of the “maximum lift-to-drag ratio”, the aerodynamic polar of the aircraft equipped with an adaptive winglet was computed using polynomial interpolation, shown on Section 2.4. In this section, the aerodynamic polar of the new and the reference configuration of the CRJ700 (fixed winglet) were compared for five Mach numbers representative of the flight envelope of the aircraft. 

Moreover, effects observed on the pitching moment are displayed in this same subsection. The pitching moment behavior is fundamental because it affects the position of the horizontal tail necessary to trim the aircraft, the limits in the variation of the Center of Gravity (CG) to avoid commands saturation or poor stability, and the flight qualities. Consequently, it was important to analyze how the pitching moment changed between the two configurations of the aircraft (fixed and adaptive winglet) in order to verify that the adaptive configuration maintains a certain stability.

**Figure 12:**
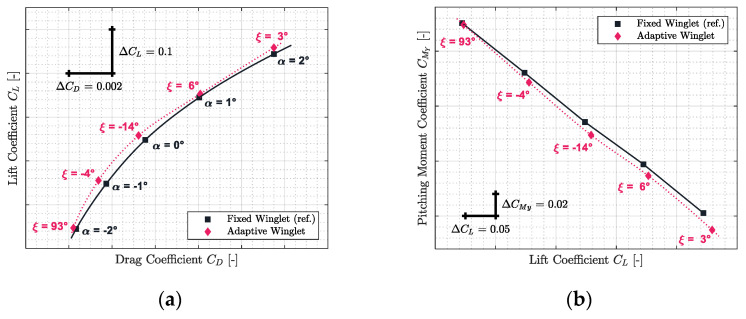
Aerodynamic polar (**a**) and pitching moment coefficient (**b**) comparison between a CRJ700 equipped with fixed and adaptive winglets at Mach number 0.31.

**Figure 13:**
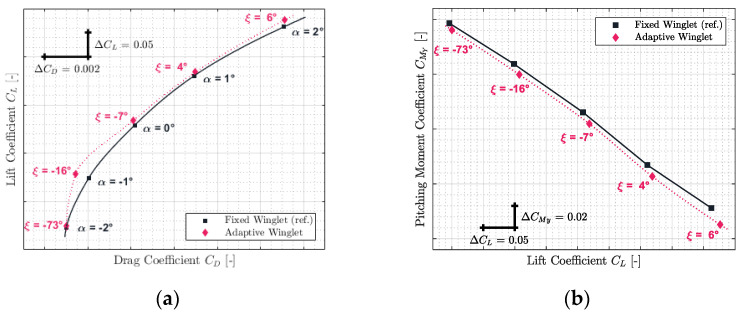
Aerodynamic polar (**a**) and pitching moment coefficient (**b**) comparison between a CRJ700 equipped with fixed and adaptive winglets at Mach number 0.45.

**Figure 14:**
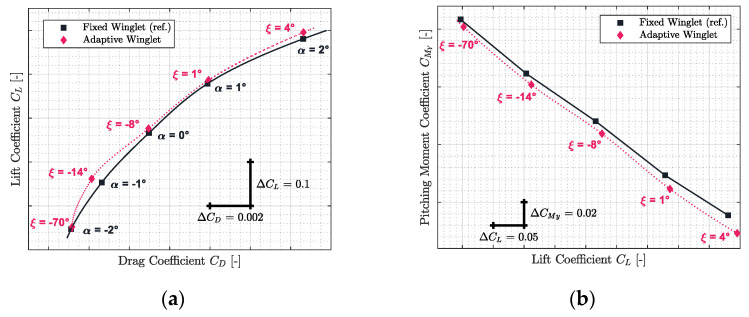
Aerodynamic polar (**a**) and pitching moment coefficient (**b**) comparison between a CRJ700 equipped with fixed and adaptive winglets at Mach number 0.54.

**Figure 15:**
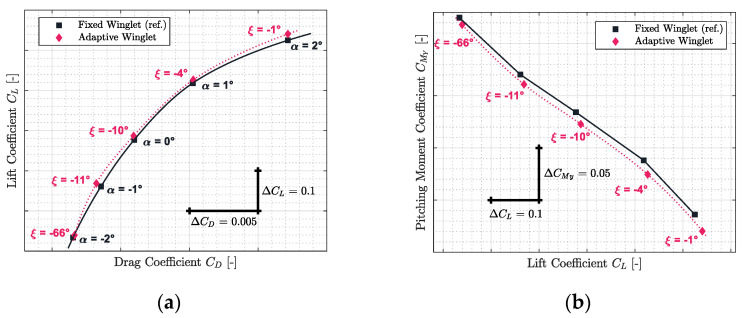
Aerodynamic polar (**a**) and pitching moment coefficient (**b**) comparison between a CRJ700 equipped with fixed and adaptive winglets at Mach number 0.66.

**Figure 16:**
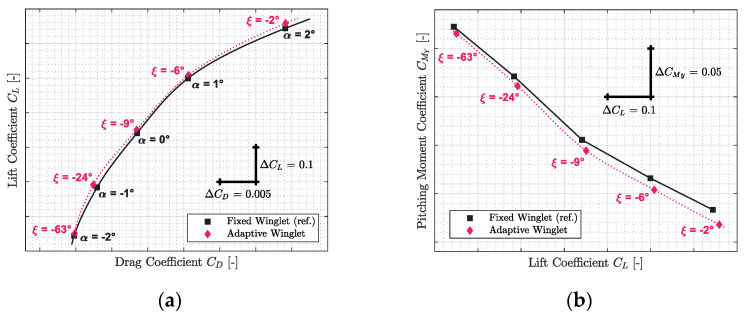
Aerodynamic polar (**a**) and pitching moment coefficient (**b**) comparison between a CRJ700 equipped with fixed and adaptive winglets at Mach number 0.79.

The results obtained for Mach numbers 0.31, 0.45, 0.54, 0.66, and 0.79 are presented in Figure 12, Figure 13, Figure 14, Figure 15 and Figure 16, respectively. Each of these figures are divided into two subfigures (a) and (b). The sub-figures (a) show the comparison in terms of aerodynamic polar between a CRJ700 aircraft equipped with a fixed (in “black” color) versus an adaptive winglet (in “pink” color). Similarly, sub-figures (b) show the comparison of the fixed versus adaptive winglets’ results in terms of the pitching moment coefficients versus lift coefficients.

It is important to add the fact that due to the discontinuous aspects of the adaptive winglet geometry (the winglet angle is changing during flight, according to the flight condition), it was preferred to first predict aerodynamic coefficients punctually and display them using “diamond” shapes markers. Then, between aerodynamic coefficients predicted, as the winglet angle could change due to the adaptive configuration, it was preferred to predict intermediate aerodynamic coefficients using spline equations and display it using “dotted” lines.

Globally, for each flight condition, it was observed in sub-figures (a) that the adaptive winglet allows the aircraft to reach an aerodynamic polar located at the left-hand side of the reference polar. Shifting the aerodynamic polar of an aircraft to the left signifies that the drag generated for a given lift has been reduced. 

Moreover, by selecting the best winglet deflection angle as the one that offered the maximum fitness to the aircraft, the pitching moment coefficient was impacted (sub-figures (b)). Indeed, it was observed that the pitching moment coefficients obtained by an aircraft equipped with an adaptive winglet were shifted to negative values (downside) with respect to their reference values (shown in “black” color). 

For example, for Mach 0.31 on Figure 12a, the aerodynamic polar corresponding to the aircraft equipped with an adaptive winglet is located on the left-hand side of the aerodynamic polar, corresponding to the aircraft equipped with the fixed winglet. For angles of attack α of 1 deg and 2 deg, “pink” markers were located on the right-hand side of the reference marker (in “black”). This signifies that, locally, for a given angle of attack, the aircraft equipped with an adaptive winglet generates more drag than with a fixed winglet. 

However, for these angles of attack (1 deg and 2 deg), “pink” markers are also located upside of the reference markers. Consequently, while there is more drag generated by an aircraft equipped with an adaptive winglet, there is also more lift. Nevertheless, it is important to combine these two characteristics of lift and drag using an aerodynamic polar. Indeed, to fly an aircraft requires a given lift that depends essentially on its weight (the angle of attack is in fact a consequence and not a requirement to maintain a cap or hold an altitude, for example). The corresponding drag is deducted by the aerodynamic polar, according to the lift required. 

From this interpretation, as the adaptive winglet polar is located on the top or on the left-hand side of the reference aerodynamic polar, it signifies that for a given lift required, the drag generated by the aircraft equipped with an adaptive winglet would be less than originally (i.e., an aircraft equipped with a fixed winglet). 

Figure 12b shows that the pitching moment coefficient obtained for the adaptive winglet configuration is approximatively reduced by 0.0067 (from 0.0061 to 0.0074) with respect to the reference value. 

From the stability point of view, original aircraft properties could be conserved. Indeed, since the slope of the pitching moment versus the lift coefficient for an adaptive winglet is similar to that of the reference, the aircraft’s neutral point of stability (aft center of gravity) has been conserved. The only difference that could be noted concerns the fact that $C_{M_{y}}$ at origin ($C_{M_{0}}$) has changed, which could affect the aircraft trim status; for instance, the trimmed angle of attack should be lower for the adaptive than for the reference configuration. There is a high probability (because the difference is small) that the $C_{M_{0}}$ difference can be completely “corrected” by the trim operation or it would slightly increase the flight speed. 

In the case where it could not be corrected by the trim operation, the fact that the $C_{M_{y}}$ was shifted could have an impact on the static margin of the aircraft and on the forward limit of its center of gravity. In this case, the slope of the pitching moment coefficient versus the angle of attack can be tuned using a change in the surface of the horizontal tail (or distance from the aerodynamic center). Indeed, the value of the pitching moment coefficient for a zero angle of attack can be finely calibrated by acting on the settling angle of the horizontal tail. An increase in the dimensions of the aircraft longitudinal control surfaces (stabilizers and/or elevators) leads to reduced control surfaces deflections to trim (or to an extension of the possible angle of attack range for which trimming is ensured). 

Practically, the same observations made for Mach number 0.31 could be made for the other flight conditions evaluated for Mach numbers 0.45, 0.54, 0.66, and 0.79. Indeed, it could be observed that in Figure 13a, Figure 14a, Figure 15a and Figure 16a, the aerodynamic polar corresponding to the aircraft equipped with an adaptive winglet (in “pink” color) is located on the left-top hand side of the reference aerodynamic polar. Moreover, these aerodynamic polars were obtained with a winglet deflection angle that is almost located within the range ξ= −25 deg to ξ= 10 deg. 

For α = −2 deg, the winglet position that offered the maximum lift-to-drag ratio is usually close to −73 deg, which is far from the others “optimal winglet deflection positions”. This could be explained by the fact that aerodynamic coefficients $C_{L}$ and $C_{D}$ obtained for α = −2 deg are very close for each winglet deflection position. Polynomial fitting surfaces (displayed in section II.D for α = 0 deg) obtained for α = −2 deg were relatively “flat” according to the winglet deflection angle axis. Indeed, if the winglet deflection was “forced” to another position, similar results could have been observed (this fact was only observed for α = −2 deg). 

### 3.3. Drag Improvement Summary

**Figure 17:**
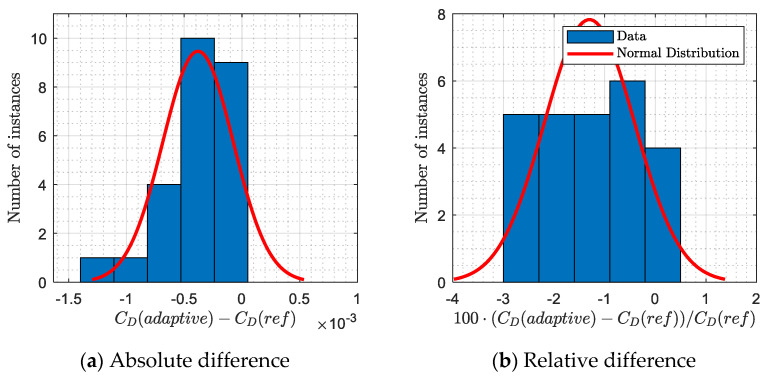
Drag benefits observed between the new aerodynamic polar (adaptive winglet) and the reference polar (fixed winglet).

This results section has been added in order to highlight the drag reduction permitted using the adaptive winglet with respect to the fixed winglet (reference). In other words, the difference in terms of drag was computed between the aerodynamic polar of the two aircraft configurations (fixed and adaptive winglet) and was displayed on Figure 17.

The drag absolute (Figure 17a) and relative (Figure 17b) differences were shown using blue bars, while the “normal” distribution of the differences was displayed using red curves. These results were obtained for the 25 flight conditions that were used to display the aerodynamic polar on Figure 12a, Figure 13a, Figure 14a, Figure 15a and Figure 16a.

Figure 17a shows that using an adaptive winglet, the drag coefficient could be reduced by up to 0.0015. In 95% of cases, the drag coefficient has been reduced by 0.00038 ± 0.00031, which corresponds to a reduction of 1.30 ± 0.89% using relative values. Moreover, Figure 17b shows that the adaptive winglet allows the drag to be reduced by up to 2.90% for several flight conditions. These encouraging results confirm that using an adaptive winglet allows the drag generated to be reduced, and therefore, it improves aerodynamic characteristics of the aircraft. As a result, a fuel burn reduction is also expected for future performance studies.

### 3.4. Evolution of the Winglet Position during a Generic Cruise Profile

In order to obtain the motion of the adaptive winglet during cruise, the optimum winglet deflection angles were chosen with respect to three optimization criteria: the maximum lift coefficient, the minimum drag coefficient, and the maximum lift to drag ratio. These optimum winglet deflections angles were found for two typical cruise profiles at Mach number 0.50 (low-speed) and Mach number 0.75 (high-speed). 

**Figure 18:**
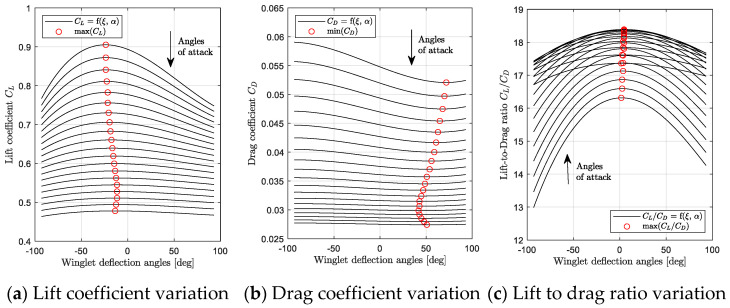
Aerodynamic characteristics variation during a generic cruise mission at Mach number 0.5 (angle of attack variation from 4 deg to 1 deg).

The results obtained for a low-speed cruise profile (Mach number 0.50) have been shown in Figure 18a–c. It was considered that at Mach number 0.50, due to the weight reduction during the flight, the angle of attack should change from 4 deg to 1 deg. In Figure 18a, it can be observed that the maximum lift coefficient was obtained when the winglet varied between −11 and −23 degrees. In Figure 18b, the minimum of drag coefficient has been obtained for winglet deflection angles from 41 to 72 deg. This range of winglet positions obtained for a “minimum drag” criterion is as large as for a “maximum lift” criterion. Finally, for an optimal criterion based on the lift to drag ratio, it could be observed on Figure 18c that the maximum fitness was obtained for a winglet position between 16 and 18 degrees.

From these observations, it could be concluded that for a low-speed cruise profile at Mach number 0.50, a winglet deflection motion from 16 to 18 degrees is required to fly using the maximum lift-to-drag criterion. If the minimum drag coefficient is preferred as the criteria for cruise optimization, the winglet deflection angle should change from 41 to 72 deg.

**Figure 19:**
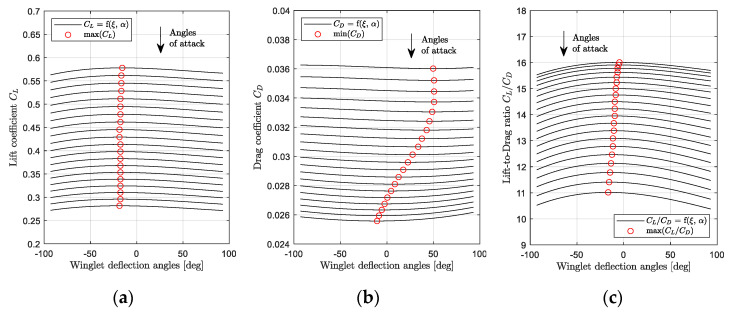
Aerodynamic characteristics variations during a generic cruise mission at Mach number 0.75 (angle of attack variation from 1 deg to −1 deg).

A similar study was conducted for a typical cruise profile at Mach number 0.75. In this case, due to the fuel burn and, therefore, to the weight reduction during the flight, it was considered that the angle of attack varied from 1 deg to −1 deg. Results expressed in terms of lift, drag, and pitching moment coefficients’ variations with winglet deflection angles are displayed in Figure 19a–c.

From Figure 19a, it was observed that the winglet angle needed to remain close to −15 degrees during the flight in order to obtain the maximum lift coefficient. However, it needed to change from −10 deg. to 50 deg. in order to offer the minimum drag coefficient along the flight (Figure 19b). Finally, to optimize the lift-to-drag ratio of the aircraft, the winglet deflection angles needed to change from −16 to −4 degrees (Figure 19c). 

More generally, by observing the general trend of the variations on these graphs, it could be supposed that the winglet position had a higher influence for low-speed cruise (Figure 18) than for high-speed cruise (Figure 19). Indeed, the curves shown in Figure 19 are flatter than those observed in Figure 18. It signifies that the aerodynamic coefficients have very close values, regardless of the winglet position. From this observation, it is expected that using an adaptive winglet, a higher improvement was achieved at low speeds than at high speeds. 

## 4. Conclusions

The research presented here is part of a larger project on the way in which an adaptive winglet can improve aircraft performances during flight. This paper has presented the aerodynamic aspects of the study applied on the Bombardier Regional Jet CRJ700. 

An adaptive winglet that has only one degree of freedom was designed from a Computer-Aided-Design (CAD 3D) model of the original CRJ700 aircraft. The adaptive winglet can move from deflection angles ξ = −93 deg to ξ = +93 deg, measured from the ground and the winglet planform. 

To compute the lift, drag, and pitching moment coefficients of this new aircraft design, a high-fidelity aerodynamic model designed using OpenFoam tools and validated using a highly qualified flight simulator (level D) was used [53]. 

A wide range of winglet deflection angles were simulated for the most commonly used flight conditions of this aircraft (i.e., for Mach numbers 0.31 to 0.79). Very promising results were obtained; for instance, the lift-over-drag ratio (i.e., the fitness) was increased by up to 6.10% by moving the winglet from −35 deg to 35 deg on the entire aircraft flight envelope. 

Using fitting polynomial surfaces, it was possible to compare the aerodynamic polar of both aircraft configurations, based on the maximum lift-to-drag ratio optimization criterion. It was observed that the aerodynamic polar of the Bombardier CRJ700 equipped with an adaptive winglet was always located on the left-hand-side of the original aerodynamic polar. This fact indicates that for a given lift coefficient required by the aircraft trim, the generated drag would be reduced. An averaged drag reduction of 1.30 ± 0.89% was predicted.

Based on these promising results, it will be interesting to realize performance studies of this aircraft equipped with adaptive winglets and verify how much fuel could be saved for typical flight profiles. It would also be interesting to investigate the benefits that could be gained by using the adaptive winglet di-symmetrically from one side to the other of the aircraft, for example, during a turn. An evaluation of the size and power of the actuation system of the adaptive wings and additional components weight could contribute to assess the usefulness of the concept developed here. Finally, an optimization design of the junction (i.e., the “pod”) and aeroelastic studies should be conducted in future studies as they were not considered in this paper due to a lack of structural data. However, it is expected that it should improve the results observed.

On another hand, it should be interesting to conduct a cost study, especially concerning the weight penalty induced by the adaptive mechanism, as well as the maximum fuel consumption that could be obtained in case of a winglet motion failure.

In spite of the maintenance costs that could be induced by such a system, the authors would like to conclude that this adaptive winglet system is very interesting for an aircraft. For this purpose, we would like to consider an example of the new generation of aircraft, equipped with oversized wingspan, requiring a folding wingtip to reach the airport facilities (such as the 777X). Indeed, since these aircrafts will be equipped with a mechanism to allow wingtip movement, the added maintenance costs to enable its motion during flight should be minimal in comparison to the aerodynamic performance improvements provided.

## Figures and Tables

**Table 1:** Average mesh qualities of validated and new aerodynamic models.

Mesh Quality Parameters	Validated Model (no Pod)	New Model (with Pod)
Max. non-orthogonality (deg)	65.060	65.020
Max. skewness	5.0006	4.9925
Number of cells (×10^6^)	11.14	11.12

**Table 2:** Flight conditions selected for the adaptive winglet study.

Altitude	Mach Number	Angle of Attack
5000 ft	M0.31	−2 deg to +2 deg
10,000 ft	M0.45	−2 deg to +2 deg
20,000 ft	M0.54	−2 deg to +2 deg
25,000 ft	M0.66	−2 deg to +2 deg
30,000 ft	M0.79	−2 deg to +2 deg
